# The impact of sex on brain responses to smoking cues: a perfusion fMRI study

**DOI:** 10.1186/2042-6410-4-9

**Published:** 2013-04-29

**Authors:** Reagan R Wetherill, Kimberly A Young, Kanchana Jagannathan, Joshua Shin, Charles P O’Brien, Anna Rose Childress, Teresa R Franklin

**Affiliations:** 1 Department of Psychiatry, University of Pennsylvania, Philadelphia, PA 19104, USA; 2 Philadelphia VA Medical Center, Philadelphia, PA 19104, USA

**Keywords:** Addiction, Sex differences, Neuroimaging, Smoking cues

## Abstract

**Background:**

Anecdotal and clinical theories purport that females are more responsive to smoking cues, yet few objective, neurophysiological examinations of these theories have been conducted. The current study examines the impact of sex on brain responses to smoking cues.

**Methods:**

Fifty-one (31 males) cigarette-dependent sated smokers underwent *pseudo-*continuous arterial spin-labeled perfusion functional magnetic resonance imaging during exposure to visual smoking cues and non-smoking cues. Brain responses to smoking cues relative to non-smoking cues were examined within males and females separately and then compared between males and females. Cigarettes smoked per day was included in analyses as a covariate.

**Results:**

Both males and females showed increased responses to smoking cues compared to non-smoking cues with males exhibiting increased medial orbitofrontal cortex and ventral striatum/ventral pallidum responses, and females showing increased medial orbitofrontal cortex responses. Direct comparisons between male and female brain responses revealed that males showed greater bilateral hippocampal/amygdala activation to smoking cues relative to non-smoking cues.

**Conclusions:**

Males and females exhibit similar responses to smoking cues relative to non-smoking cues in *a priori* reward-related regions; however, direct comparisons between sexes indicate that smoking cues evoke greater bilateral hippocampal/amygdalar activation among males. Given the current literature on sex differences in smoking cue neural activity is sparse and incomplete, these results contribute to our knowledge of the neurobiological underpinnings of drug cue reactivity.

## Background

Smoking cessation treatments are efficacious in some smokers, but overall treatment success is modest (20-40%), reflecting heterogeneity in treatment response [1]. In an attempt to improve cessation outcomes, research has started to focus on individual differences that contribute to cigarette smoking behavior and treatment response. One factor that may influence smoking maintenance and treatment outcome is the smoker’s sex. Although smoking rates have declined over the past 40 years, the rate of decline among women has been less than the rate of decline among men (20% compared to 30%, respectively; [2]). The mechanisms underlying why women have lower rates of cessation than men remain unknown; however, some research suggests that sex differences in responses to nicotine and non-nicotine factors may influence smoking cessation outcomes [3,4].

Both preclinical and clinical literature indicate that once nicotine/cigarette dependence is established, smoking behavior in males may be influenced more by maintaining nicotine levels in the brain; whereas, female smoking behavior may be influenced more by non-nicotine factors, such as smoking-related stimuli (i.e., smoking cues (SCs)) (for a review see [5]). For example, males are more accurate at discriminating variations in the amount of nicotine in cigarettes and spray than females [6,7]. Females, however, show increased smoking reinforcement relative to males, as measured by cigarette puffs, when exposed to a lit cigarette cue [8]. In a subsequent study, Perkins and colleagues found that females showed decreased *ad lib* puff self-administration and hedonic ratings of puff self-administration relative to males when olfactory and gustatory SCs were blocked (i.e., blocking nostrils while smoking cigarettes via nose-clips) [9]. In other work, it was shown that females received more craving relief from smoking a denicotinized cigarette than males [10]. Together, these findings suggest that non-nicotinic aspects of smoking influence females’ smoking behavior more than males.

The picture becomes less clear in studies examining the impact of sex on physiological and subjective responses to SCs [11-14]. In some studies, females reported higher SC-induced subjective craving and showed greater changes in mean arterial pressure than males [11,13], yet Tong and colleagues [12] found that males had higher blood pressure and skin temperature responses to SCs than females. Other studies, however, reported no sex differences in physiological or subjective reports of craving [14,15]. While these inconsistent findings may be related to methodological differences between studies, they may also be related to caveats associated with self-report and physiological assessments. Thus, the current study uses an objective, neurobiological approach to examine the impact of sex on brain responses to SCs.

Previous neuroimaging studies examining neural responses to SCs have consistently shown robust brain responses to SCs in reward-related mesocorticolimbic circuitry (medial orbitofrontal cortex (mOFC), ventral striatum/ventral pallidum (VS/VP), hippocampus, amygdala, and insula) [16-19]. Although cigarette-dependent smokers exhibit characteristic neural responses to SCs, the impact of sex on neural responses to SCs remains largely unexplored. One functional magnetic resonance imaging (fMRI) study found that females exhibited SC-induced brain responses in the cuneus and superior temporal gyrus, while males showed brain responses in hippocampus and orbitofrontal cortex [20]; however, sample size was small (females: *n* = 7) and direct comparisons of SC-induced brain responses between females and males were not explored. In fact, no neuroimaging studies to date have directly compared male and female brain responses to SCs.

To this end, the current study directly investigated the effects of sex on brain responses to SCs. Given that males and females respond differently to nicotine and nicotine-related factors, we hypothesized that they would also show differences in brain responses during SC exposure. Based on previous research suggesting greater behavioral and physiological responses to SCs among females relative to males [8,9,11,13], we hypothesized that females would show greater brain responses to SCs than males in brain regions that we have consistently shown to be associated with SC-reactivity (i.e., mOFC, VS/VP, hippocampus, amygdala, and insula) [16,21-23]. To test this hypothesis, we used the technique of *pseudo-*continuous arterial spin-labeled (*p*CASL) perfusion fMRI to acquire brain responses during SC (versus non-SC) exposure.

## Methods

### Participants

Participants were recruited via radio advertisements and local list-serves describing a study for smokers contemplating quitting, but not ready to quit. Telephone screens, as well as medical and psychiatric evaluations were used to determine participant eligibility. Ineligible participants were those who reported other current substance dependence, had current Axis I DSM IV psychiatric diagnoses, had significant medical conditions, reported a history of head trauma or injury causing loss of consciousness lasting greater than three minutes or associated with skull fracture or intracranial bleeding, or had irremovable magnetically active objects on or within their body. All eligible and interested participants provided informed consent prior to their inclusion in the study.

Fifty-one physically healthy smokers (31 males) ranging in age from 18 to 58 years (34.2 ± 11.5) participated in the study. The sample is comprised of 69% Caucasians, 22% African Americans, and 9% Other/Mixed race. Perfusion fMRI data from these participants were previously reported in a study examining genetic influences on SC responses [23]. Following consent, participants completed psychological and physical evaluations. Participants received $100.00 for completing the study. The study adhered to the Declaration of Helsinki and was approved by the University of Pennsylvania Institutional Review Board.

### Measures

The MINI International Neuropsychiatric Interview [24] assessed current DSM-IV diagnosis of substance dependence other than nicotine and current severe psychiatric symptoms. The Fagerstrom Test for Nicotine Dependence (FTND) [25] assessed severity of nicotine dependence. The Craving and Withdrawal Questionnaire (CWQ) measured subjective ratings of craving, withdrawal, mood state, and interest in the video before and after stimulus presentations during the scan session.

### Imaging approach

*Pseudo*-continuous arterial spin-labeled (*p*CASL) perfusion fMRI, a quantitative estimate of cerebral blood flow (CBF) and indirect measurement of neural activity [26], assessed brain activation in response to SC exposure. Before the scanning session, participants smoked *ad lib* to minimize nicotine withdrawal-induced craving that might accrue during the scanning session. Scanning occurred approximately 25 minutes after smoking to allow the acute cardiovascular effects of smoking to dissipate. During each scanning session, participants completed, in sequence, a five minute resting baseline scan; a 10 minute non-SC *p*CASL scan; a high resolution structural scan; and a 10 minute SC *p*CASL scan.

Ten-minute audio-visual clips were presented during *p*CASL scanning. The SC video included individuals differing in race, age, and sex who were smoking and using explicit language designed to induce appetitive desire for a cigarette. The non-SC video was similar in content; however, the video did not portray cigarette smoking or smoking reminders. The non-SC video was shown before the SC video to minimize interference in ‘carryover’ arousal initiated when drug cues are shown first, which can potentially affect responses to nondrug cues [27-29].

### Imaging data acquisition

Imaging data were acquired on a 3.0 Tesla Trio whole-body scanner (Siemens AG, Erlangen, Germany) using a Bruker volume coil (volume coils are designed to provide a homogenous receiving sensitivity and are 1 channel; Bruker Biospin, Billerica, MA) for 19 subjects and a standard 8-channel receive-only array head coil for the remaining 32 subjects. For co-registration of the functional data, a T1-weighted three-dimensional (3D) high resolution MPRAGE scan was acquired with field of view (FOV) = 160 mm, repetition time (TR) = 1510 ms, echo time (TE) = 3 ms, 192 × 256 matrix, slice thickness 1 mm for 19 subjects and FOV = 250 mm, TR/TE = 1620/3 ms, 192 × 256 matrix, slice thickness 1 mm for the remaining 32 subjects. *p*CASL perfusion fMRI sequence was used for resting baseline, SC and non-SC data acquisition. Interleaved images with and without labeling were obtained using a gradient echo echo-planar imaging sequence with a delay of 1000 ms for 19 subjects or 700 ms for 32 subjects inserted between the end of the labeling pulse and image acquisition (FOV = 130 mm, matrix = 64 × 64 × 14, TR/TE = 3000/17 ms, flip angle = 90°, slice thickness = 6 mm with a 2 mm inter-slice gap for 32 subjects and a 1.2 mm inter-slice gap for 19 subjects.

### Imaging data processing and statistical analyses

An SPM-based arterial spin labeling (ASL) data processing toolbox [30] was used for *p*CASL perfusion data analyses, as described previously [21,22]. Briefly, ASL image pairs were realigned to the mean of all control images and spatially smoothed with a 3D isotropic Gaussian kernel at 10 mm full width at half maximum (FWHM). For both SC and non-SC stimuli, 100 CBF image series were generated from the 100 label/control ASL image pairs using a simplified two-compartment model with the sinc interpolation method for CBF calculations [31]. For resting baseline (RB), 48 CBF image series were generated from the 48/label/control ASL image pairs using the same methods for CBF calculations. The mean control image of each subject’s data was co-registered to the structural image using the mutual information based co-registration algorithm provided by SPM8. The same transformation parameters were applied to co-register the CBF maps to each subject's anatomical image. Subsequently, the structural image was spatially normalized to the Montreal Neurological Institute (MNI) standard brain. The resulting transformation matrix was used to align the CBF images to MNI space. A binary brain mask was used to exclude the non-brain areas in the CBF maps.

Contrasts between SC versus non-SC sets were defined in the general linear model (GLM) model to assess the voxel by voxel CBF difference for each subject. Using the corresponding parametric maps of the contrast, random effects analysis was employed to test for a significant main effect of condition (SC versus non-SC) in each sex with a statistical parametric map of the *T*-statistic at each voxel for population inference within our regions of interest (ROI) mask. Based on our previous studies on SC-reactivity among cigarette-dependent smokers [16,21-23], the ROI mask included the mOFC, VS/VP, hippocampus, extended amygdala (i.e., amygdala; bed nucleus of stria terminalis (BNST)), anterior cingulate cortex (ACC), and insula. The ROI mask was created using the Harvard–Oxford probabilistic anatomical atlas provided with FMRIB Software Library (FSL) [32] and is available for viewing at http://franklinbrainimaging.com/. Significant voxels passed a voxelwise statistical threshold (*p* < 0.005) and, to control for multiple comparisons, were required to be part of a cluster > 54 voxels, as determined by a Monte-Carlo simulation and resulted in 5% probability (corrected) of a cluster surviving due to chance. Brain and behavioral correlates were examined using linear regression analyses for each sex using change in craving scores to SCs and brain activity at each voxel within the ROI mask.

### Post hoc analyses

Post hoc analyses examined potential sex differences in resting baseline by extracting CBF from the significant functional clusters within the ROI mask wherein SCs elicited greater neural activity (i.e., mOFC, VS/VP, and hippocampus/amygdala). The average quantitative CBF values (ml of blood/100 g of tissue/minute) was computed for females and males and then compared with independent samples *t*-tests. Additional post hoc analyses examined sex differences in brain responses to non-SCs to determine whether females and males differed in brain responses to visual cues, in general. This analysis used the same GLM and random effects analysis described above; however, the analysis used an exploratory whole-brain approach and the non-SC versus resting baseline contrast was used rather than the SC versus non-SC contrast.

### Covariates

Consistent with recent national survey data indicating higher rates of cigarette use among males than females [33], males in the current study smoked more cigarettes per day than females. Therefore, the cigarettes smoked per day variable was included in analyses as a covariate.

### Demographic and behavioral statistical analyses

Continuous demographic variables were summarized, by calculating means and standard error measurements (X ± SEMs). For the CWQ data, change scores were calculated as follows: post-SC video score – pre-SC video score. Independent samples *t*-tests compared females and males on continuous variables. Nominal demographic variables were summarized by calculating proportions and compared across groups using chi-square analyses. A repeated measures analysis of variance (ANOVA) with time (i.e., pre- and post-SC ratings) as the within-subjects factor and sex (i.e., male or female) as the between subject factor was used to determine the effects of SC exposure on subjective ratings of craving, feeling calm, feeling content, and the need to smoke a cigarette for relief.

## Results

### Participant characteristics

Table 1 provides participant demographics and smoking history characteristics. There were no significant sex differences in years of education, age, race, number of years smoking, or FTND scores (*p*s > 0.10). Sex differences emerged for cigarettes smoked per day, *t*(49) = 2.12, *p* = 0.04) and pack years (a measure to quantify intensity of chronic cigarette exposure since smoking initiation), *t*(49) = 2.11, *p* = 0.04, with males having higher values on these measures than females. Participants smoked 15.6 ± 0.8 cigarettes per day, and FTND scores were 4.5 ± 0.2, indicating moderate nicotine dependence.

**Table 1:** Participant characteristics

	**All**	**Males**	**Females**	***p***
	***N*** **= 51**	***n*** **= 31**	***n*** **= 20**	
Race (%)				
White	35 (69)	22 (71)	13 (65)	0.60
Black	11 (22)	7 (23)	4 (20)	
Other	5 (9)	2 (6)	3 (15)	
Means ± (SEMs)				
Age	34.2 (1.6)	36.2 (2.0)	30.9 (2.5)	0.10
Education	14.4 (0.3)	14.3 (0.3)	14.7 (0.6)	0.52
Cigarettes per day	15.6 (0.8)	16.9 (1.0)	13.6 (1.2)	0.04*
Pack years^a^	12.5 (1.6)	15.2 (2.4)	8.4 (1.6)	0.04*
FTND scores	4.5 (0.2)	4.7 (0.3)	4.3 (0.5)	0.44
Craving scores^b^	0.8 (0.2)	0.8 (0.3)	0.9 (0.4)	0.93

* *p* < 0.05.

^a^Pack years calculation: Cigarettes per day (÷) cigarettes in a pack (X) years smoking.

^b^Craving scores calculation: Post-SC video craving score – Pre-SC video craving score.

FTND = Fagerstrom Test for Nicotine Dependence; FTND scores ranged from 1 to 9.

### Subjective ratings

A repeated measures ANOVA revealed main effects of time (*F*_1,49_ = 23.79, *p* < 0.001, η^2^ = 0.33) and sex (*F*_1,49_ = 5.89, *p* = 0.02, η^2^ = 0.11) with an overall increase in subjective reports of craving for a cigarette following exposure to SCs and females reporting greater craving than males. Time x Sex interaction was not significant (*p* = 0.94) indicating similar increases in craving following SC exposure for females and males (Table 2-item 3). There was a significant main effect of time (*F*_1,49_ = 14.63, *p* < 0.001, η^2^ = 0.23) in subjective reports of a need to smoke for relief following exposure to SCs with overall increases in the need to smoke for relief following SC exposure. The main effect of gender and the Time x Sex interaction were not significant. No significant main effects or interactions emerged for the other items of the CWQ, which assessed the extent to which the participant felt calm and content. An independent *t*-test examined sex differences in interest in the SC video and revealed no significant differences (*p* = 0.97) (Table 2).

**Table 2:** Craving and withdrawal questionnaire items

	**Females**	**Males**	***p***
1) How calm are you right now?	−0.15 ± 0.99	0.00 ± 1.32	0.67
2) How content do you feel right now?	0.15 ± 0.99	−0.16 ± 0.93	0.26
3) How much do you desire a cigarette right now?	1.00 ± 1.81	0.97 ± 1.08	0.94
4) How much do you need to smoke right now for relief?	0.55 ± 1.67	1.03 ± 1.28	0.25
5) How interested were you in the video you just watched?	3.40 ± 1.88	3.42 ± 1.95	0.97

For items 1–7, scores were generated from the difference between pre-smoking cue exposure to post-smoking cue exposure. Each item was inserted into the phrase “On a scale from 1 to 7….. with 1 corresponding to *Not at all* and 7 corresponding to *Extremely*”.

### Imaging results

#### Smoking cue reactivity in males and females

**Figure 1:**
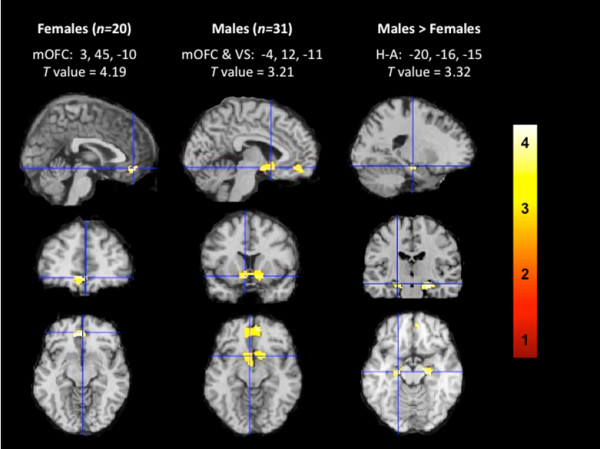
**Brain responses to smoking cues relative to non-smoking cues.** For females crosshairs are centered on the medial orbitofrontal cortex (mOFC), for males crosshairs are centered on the ventral striatum (VS), and for direct comparisons between males and females crosshairs are centered on the left hippocampus/amygdala (H-A). Representative fMRI sagittal, axial, and coronal brain slices analyzed in SPM8 and overlain on the MNI brain. T values range from 3.10 to 4.19, corrected at *p* < 0.005. Images are displayed neurologically (left is left). An interactive visual display of all brain data in all three planes can be found at http://franklinbrainimaging.com.

**Figure 2:**
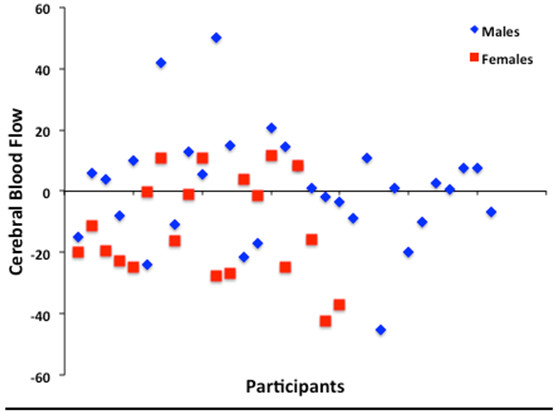
**Cerebral blood flow in the left hippocampus/amygdala during smoking cue exposure.** Cerebral blood flow in the left hippocampus/amygdala (H-A) during smoking cue exposure in male and female smokers. Cerebral blood flow = ml of blood/100 g of tissue/minute.

Males and female sated-smokers exhibited similar brain responses to SCs relative to non-SCs, with males exhibiting greater activity in mOFC and VS/VP regions and females showing enhanced activity in the mOFC. Comparisons between groups revealed significant sex differences with males exhibiting greater SC-induced brain activity in bilateral clusters spanning hippocampal and amygdalar regions compared with females (Figures 1, 2). An interactive visual display of all brain data and unmasked data at a reduced threshold can be found at http://franklinbrainimaging.com.

#### Brain and craving score correlates by sex

Using a corrected voxelwise statistical threshold (*p* < 0.005 and cluster size > 54), analyses revealed that SC-elicited craving scores (post-SC video – pre-SC video) did not correlate with SC-induced brain activity for males or females. Overall craving scores (pre- or post-SC video) also did not correlate.

#### Post hoc analyses

Given that direct comparisons between males and females revealed that males exhibited greater responses to SCs in the hippocampus/amygdala compared with females and this finding is in opposition to our hypothesis, we hypothesized that higher resting baseline CBF in females may be interfering with the ability to capture SC-induced signal. Thus, in post hoc analyses, CBF values were extracted from the functional ROIs wherein SCs elicited greater activity than non-SCs (i.e., VS, mOFC and hippocampus/amygdala). Females had significantly greater resting baseline CBF than males in the VS (50.59 ml of blood/100 g of tissue/minute in females versus 40.94 ml blood/100 g tissue/min in males; *p* = 0.01) and the mOFC (68.50 ml blood/100 g tissue/min in females versus 53.82 ml blood/100 g tissue/min in males; *p* = 0.001), but females and males did not differ in resting baseline CBF in the hippocampus/amygdala (55.57 ml blood/100 g tissue/min in females versus 50.40 ml blood/100 g tissue/min in males; *p* = 0.174). These values were then entered into the SC vs non-SC contrast as covariates to determine whether SC results were obscured, facilitated or independent of resting baseline CBF. Results were unchanged in all analyses (Males: VS and mOFC; Females: mOFC; and Males vs Females: bilateral hippocampus/amygdala).

In an attempt to shed light on potential mechanisms underlying the impact of sex on brain responses to SCs, additional exploratory *post hoc* analyses examined sex differences in brain responses to non-SCs to determine whether females and males differed in brain responses to visual cues in general. No differences between groups were observed.

## Discussion

To our knowledge, this is the first neuroimaging study to directly examine sex differences in brain responses to SCs by explicitly comparing male and female brain activity to SCs relative to non-SCs. As expected, both male and female sated-smokers showed greater brain activation to SCs relative to non-SCs. Males exhibited SC-induced brain activity in the mOFC and VS/VP, and females showed activation in the mOFC. We also hypothesized that females would show greater brain responses to SCs than males; however, direct comparisons revealed that males showed greater SC-induced brain activity than females in bilateral clusters spanning hippocampal/amygdala regions.

We observed increased brain activity to SC vs non-SCs in the mOFC of male and female smokers and increased neural activity to SCs in the VS/VP among males. These findings are analogous to our earlier studies showing mOFC and VS/VP activity in response to SCs [16,21,22]. The mOFC and VS/VP are functionally related regions involved in the processing of the incentive salience of rewards and reward-related cues [34]. Specifically, the mOFC has been consistently implicated in reward processes [35,36], and as such, it is not surprising that SCs evoke greater mOFC responses than non-SCs among smokers. Males also showed enhanced brain activity to SCs in the VS/VP. Neuroimaging evidence suggests that the VS/VP is associated with reward anticipation [37], subsequent reward-related behavior [38], and reward consumption [39]. Enhanced VS/VP activity during SC- relative to non-SC exposure perhaps suggests that viewing SCs evoked greater anticipation and reward than viewing non-SCs. Of note, females also showed increased VS response to SCs relative to non-SCs at a more liberal threshold (see http://franklinbrainimaging.com), but this increased activation did not reach significance using our conservative analytic approach.

Although males and females showed reward-related activation to SCs, direct comparisons between SC-induced brain responses revealed sex-specific differences. Males exhibited greater brain responses to SCs in bilateral clusters spanning portions of the hippocampus and amygdala, but there were no areas in which females showed greater brain responses to SCs relative to males. The hippocampus and amygdala are structures associated with emotional and drug memories [40,41], and as such, our findings may be explained by memory-related sex differences evoked during the 10-minute smoking cue video. Specifically, research indicates that females recall personally experienced events (i.e., autobiographical memories) faster and better than males, especially when those memories are emotionally relevant [42,43]. In other words, females appear to be more efficient at retrieving autobiographical memories, and therefore, males may require greater memory-related, hippocampal-amygdalar brain activity when retrieving cue-associated memories. Although this interpretation is speculative and an abductive inference, recent evidence reporting that males show greater hippocampal activation than females when recalling autobiographical memory, such as smoking a cigarette, supports this interpretation [44].

This differential finding may also be partially explained by variations in menstrual cycle phase/circulating gonadal hormones (e.g., estradiol and progesterone) among female participants. Preclinical and clinical evidence indicates that menstrual cycle phase plays an important modulatory role in reward-related brain and behavioral responses in females [45,46]. Women in the luteal phase demonstrate differential activation in brain regions involved in the response to reward (e.g., OFC, VS, insula, hippocampus, amygdala) during reward-related tasks as compared to women in the follicular phase [46,47]. Further, circulating levels of estrogen and progesterone, which vary throughout the menstrual cycle, correlate with brain activity in these regions [47]. Consequently, cyclic changes in neural responsiveness due to menstrual cycle phase/circulating gonadal hormones may influence sex differences in mesocorticolimbic brain activity. Indeed, in a recent neuroimaging study that examined neural activity in response to negative visual stimuli, no significant differences in brain activity were noted between males and females when females were scanned in the early follicular phase; however, differences emerged in reward-related brain regions between males and these same females when they were scanned around the time of ovulation [48]. Collectively, these studies indicate that menstrual cycle phase may influence the impact of sex on brain response to visual cues.

Correlative relationships between brain response and craving were not observed. One contributing factor may be the stringent thresholding criteria utilized in this study. Another contributing factor could be variations in how participants labeled their craving. Subjective reports of craving in drug-dependent populations are fraught with caveats, including difficulty in labeling emotions [49]. Indeed, several studies demonstrating correlations between craving induced by drug cues and brain activity are often in opposition [16,18,21,22,50] or have not been observed [51-54]. The findings reported here and the lack of agreement among myriad studies highlight the limitations associated with subjective measures and encourage the use of objective markers.

The current findings should be interpreted in light of the following limitations. A potential confound in our findings could be due to differences in data acquisition. For example, the first 19 subjects were scanned using a Bruker coil; whereas, the remaining subjects were scanned using an 8-channel coil. To explore whether data acquisition differences affected findings, we compared variances between both groups using a homogeneity of variance test and found that the variances were not significantly different. This study is also limited in that we focused solely on sex differences in brain responses to SCs. We recognize that many factors are at play, including menstrual cycle, negative affect/mood, stress, and variance in genes [22,55-57]. Specifically, in our lab we have found and confirmed a robust effect of dopamine transporter genotype on neural responses to SCs [21,22]. We are confident the results reported here are unrelated to dopamine transporter genotype because there is equivalent representation of the variants between males and females. As noted above, we also have evidence-based hypotheses regarding the role of menstrual cycle phase/gonadal hormones on female response to SCs. To date, however, our sample sizes are insufficient to assess the influence of menstrual cycle phase/gonadal hormones. Of the 20 female participants, 10 females were taking oral contraceptives, two were postmenopausal, six were in the follicular phase, and two were in the luteal phase. We continue to acquire data in order to obtain sample sizes that are sufficient to assess this, as well as the main effects and interactions of other factors on relapse vulnerabilities.

## Conclusions

In summary, anecdotal and clinical theories purport that females are more responsive to SCs, yet few objective, neurophysiological examinations of these theories have been conducted. The current study is the first neuroimaging study to directly compare male and female brain responses to SCs. Overall, male and female sated-smokers showed similar activation to SCs, with males showing greater brain activity in the mOFC and VS/VP during SC exposure relative to non-SC exposure, and females exhibiting greater activation in the mOFC. Direct comparisons between male and female brain responses to SCs relative to non-SCs revealed that males showed greater bilateral hippocampal/amygdalar activation to SCs than females, which may be explained by sex differences in memory processes elicited by cue exposure and variations in menstrual cycle phase/circulating gonadal hormones. The primary findings of SC reactivity modulated by sex, in *a priori* reward-related regions supported by both clinical and preclinical studies, has been underexplored, and thus, the findings contribute to our knowledge of the neurobiological underpinnings of drug cue reactivity.

## Abbreviations

ACC: Anterior cingulate cortex; ASL: Arterial spin labeling; BNST: Bed nucleus of stria terminalis; CBF: Cerebral blood flow; CWQ: Craving and withdrawal questionnaire; fMRI: functional magnetic resonance imaging; FOV: Field of view; FSL: FMRIB software library; FTND: Fagerstrom test for nicotine dependence; FWHM: Full width at half maximum; GLM: general linear model; MINI: Mini international neuropsychological interview; MNI: Montreal neurological institute; mOFC: medial Orbitofrontal cortex; MPRAGE: Magnetization-prepared rapid gradient echo; pCASL: pseudo-continuous arterial spin labeling; RB: Resting baseline; ROI: Region of interest; SC: Smoking cue; TE: Echo time; TR: Repetition time; VS/VP: Ventral striatum/ventral pallidum

## Competing interests

Dr. O’Brien has received compensation as a consultant for Alkermes and Reckitt Benckiser. All other authors declare that they have no competing interests.

## Authors’ contributions

RRW drafted the manuscript and performed statistical analyses. KA assisted in drafting the manuscript. KJ analyzed the neuroimaging data. JS assisted in data collection. TRF conceived the study, assisted in manuscript preparation, and participated in its design and coordination. All authors read and approved the final manuscript.
